# Model-based Vestibular Afferent Stimulation: Modular Workflow for Analyzing Stimulation Scenarios in Patient Specific and Statistical Vestibular Anatomy

**DOI:** 10.3389/fnins.2017.00713

**Published:** 2017-12-19

**Authors:** Michael Handler, Peter P. Schier, Karl D. Fritscher, Patrik Raudaschl, Lejo Johnson Chacko, Rudolf Glueckert, Rami Saba, Rainer Schubert, Daniel Baumgarten, Christian Baumgartner

**Affiliations:** ^1^Department for Biomedical Computer Science and Mechatronics, Institute of Electrical and Biomedical Engineering, University for Health Sciences, Medical Informatics and Technology, Hall in Tirol, Austria; ^2^Department for Biomedical Computer Science and Mechatronics, Institute of Biomedical Image Analysis, University for Health Sciences, Medical Informatics and Technology, Hall in Tirol, Austria; ^3^Department of Otolaryngology, Medical University of Innsbruck, Innsbruck, Austria; ^4^Department of Otolaryngology Tirol Kliniken, University Clinics Innsbruck, Innsbruck, Austria; ^5^MED-EL GmbH, Innsbruck, Austria; ^6^Department of Computer Science and Automation, Institute of Biomedical Engineering and Informatics, Technische Universität Ilmenau, Ilmenau, Germany; ^7^Faculty of Computer Science and Biomedical Engineering, Institute of Health Care Engineering, Graz University of Technology, Graz, Austria

**Keywords:** vestibular implant, virtual model, optimization, modular workflow, nerve fibers, potential distribution, finite element model, human anatomy

## Abstract

Our sense of balance and spatial orientation strongly depends on the correct functionality of our vestibular system. Vestibular dysfunction can lead to blurred vision and impaired balance and spatial orientation, causing a significant decrease in quality of life. Recent studies have shown that vestibular implants offer a possible treatment for patients with vestibular dysfunction. The close proximity of the vestibular nerve bundles, the facial nerve and the cochlear nerve poses a major challenge to targeted stimulation of the vestibular system. Modeling the electrical stimulation of the vestibular system allows for an efficient analysis of stimulation scenarios previous to time and cost intensive *in vivo* experiments. Current models are based on animal data or CAD models of human anatomy. In this work, a (semi-)automatic modular workflow is presented for the stepwise transformation of segmented vestibular anatomy data of human vestibular specimens to an electrical model and subsequently analyzed. The steps of this workflow include (i) the transformation of labeled datasets to a tetrahedra mesh, (ii) nerve fiber anisotropy and fiber computation as a basis for neuron models, (iii) inclusion of arbitrary electrode designs, (iv) simulation of quasistationary potential distributions, and (v) analysis of stimulus waveforms on the stimulation outcome. Results obtained by the workflow based on human datasets and the average shape of a statistical model revealed a high qualitative agreement and a quantitatively comparable range compared to data from literature, respectively. Based on our workflow, a detailed analysis of intra- and extra-labyrinthine electrode configurations with various stimulation waveforms and electrode designs can be performed on patient specific anatomy, making this framework a valuable tool for current optimization questions concerning vestibular implants in humans.

## 1. Introduction

**Figure 1:**
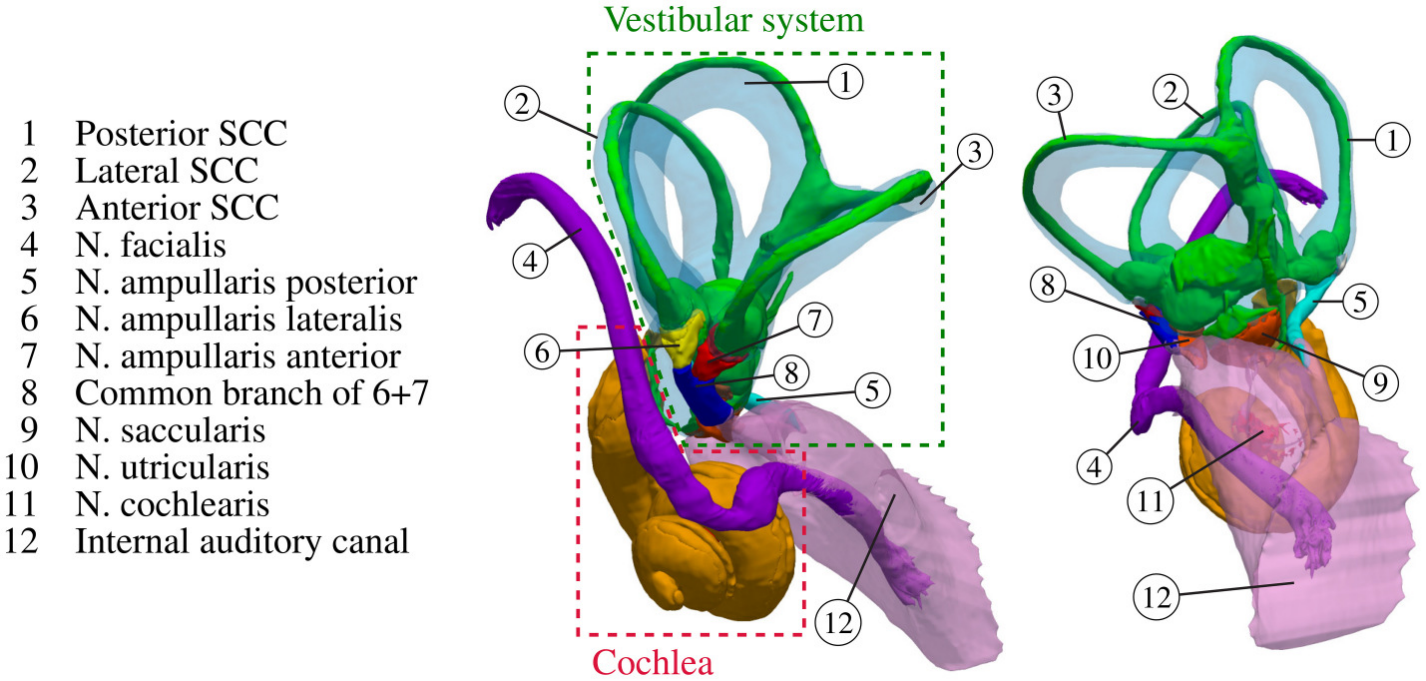
Anatomy of the inner ear with highlighted semicircular canals and vestibular nerves. The bony labyrinth is depicted as a transparent blue volume containing the segmented endolymph in green.

Our sense of balance and spatial orientation strongly depends on the correct functionality of our vestibular system. It contributes to the stabilization of gaze during head motion through the vestibulo-ocular reflex and to postural control and spatial orientation by other pathways (Nguyen et al., 2016). Figure 1 depicts the anatomy of the inner ear. While rotational motion is primarily sensed by the sensory epithelia in the ampullae of the three semicircular canals (SCC), horizontal and vertical acceleration is perceived by the two otolith organs, the utricle and the saccule. Five nerve branches that innervate these structures [nervus (N.) ampullaris posterior, N. ampullaris anterior, N. ampullaris lateralis, N. utricularis, N. saccularis] conduct the stimuli from the sensory epithelia to vestibular centers in the brain. The vestibular nerve branches merge with the N. cochlearis to form the vestibulocochlear nerve, which travel together with the N. facialis through the internal auditory canal (IAC) to the brainstem (Khan and Chang, 2013). The vestibular ganglion neurons innervating distinct sensory epithelia show a unique and only partial overlapping distribution in the superior and inferior vestibular ganglion and rotate along their longitudinal axis within the IAC (Sando et al., 1972). A study of Maklad et al. (2010), who traced neural paths from the brainstem and cerebellum with fluorescent lipid soluble dyes in mice, showed that the vestibular nerve branches, the N. cochlearis and the N. facialis running within the IAC enter distinct nuclei in the brain and that vestibular fibers project either to the cerebellum or the brainstem depending on the location of their origin at the corresponding sensory epithelium.

Vestibular dysfunction can lead to blurred vision and impaired balance and spatial orientation, causing a significant decrease of quality of life (Guinand et al., 2012; Sun et al., 2014; van de Berg et al., 2017). While unilateral and mild to moderate bilateral vestibular dysfunction can be (partially) compensated by the remaining function of the vestibular system and other sensory input in most cases, insufficient compensation on severe bilateral vestibular dysfunction can be devastating (Sun et al., 2014). Causes of bilateral vestibular dysfunction include ototoxic effects, genetic abnormalities, Ménière's disease, labyrinthitis, meningitis, ischemia autoimmune disease, and idiopathic or iatrogenic injury (Sun et al., 2014). Age-dependent deterioration of vestibular function is evident, which is caused by “significant degeneration in nearly all types of vestibular cells, including the sensory end organ hair cells, the nerve fibers, Scarpa ganglion cells, vestibular nucleus neurons, and even a significant decline in the number of Purkinje cells within the cerebellum” (Zalewski, 2015). Vestibular implants offer a possible treatment option for patients with vestibular dysfunction. By selective stimulation of vestibular nerve fibers based on the input of motion sensors of the implant, vestibular function can be (partially) restored.

Animal experiments were undertaken to prove the feasibility of vestibular implants (Della Santina et al., 2007; Fridman and Della Santina, 2012; Rubinstein et al., 2012; Nie et al., 2013; Lewis, 2016) and also to analyze the effects of different stimulation parameters on the stimulation of the vestibular nerve (Fridman et al., 2010; Davidovics et al., 2011). The feasibility of vestibular implants was also confirmed in humans (Guyot et al., 2011; Perez Fornos et al., 2014; Guinand et al., 2015; van de Berg et al., 2017). The close proximity of the vestibular nerve bundles, the N. facialis and the N. cochlearis poses a major challenge in targeted stimulation of the vestibular system. Nerve branches not targeted for stimulation may be spuriously activated by current spread and imprecise electrode positioning, leading to a misaligned perception of acceleration (Davidovics et al., 2011; Hayden et al., 2011; Marianelli et al., 2015).

Virtual models of electrical stimulation of the vestibular system were developed to allow for optimization of the electrode design, positioning and stimulus waveforms prior to time and cost intensive *in vivo* experiments. Hayden et al. (2011) developed a virtual model of the vestibular system of chinchillas and tested the effects of different electrode configurations, stimulus waveforms and amplitudes using spherical electrodes positioned inside the bony labyrinth. Their results were validated by comparing the angular vestibulo-ocular reflex measured during *in vivo* experiments with corresponding simulated results. Marianelli et al. (2015) used a similar approach on a CAD volume model based on human anatomy to test extra-labyrinthine electrode configurations. Changes in selectivity and the effect on the vestibulo-ocular reflex were analyzed based on variation of monopolar electrode configurations close to the corresponding target nerves.

Although these virtual models showed promising results in the evaluation and optimization of vestibular stimulation scenarios, the underlying volume models are currently either based on animal data or simplified human anatomy, in which either intra- or extra-labyrinthine electrode configurations were investigated. Additional *in silico* experiments using virtual models based on more realistic human anatomy could increase the confidence in the results obtained by the existing frameworks and lead to new findings for the optimization of (patient specific) vestibular stimulation strategies.

In the course of the *Vestibular Anatomy Modeling and Electrode Design* (VAMEL) project, μCT scans of human vestibular specimens were obtained (Johnson Chacko et al., submitted), which served as a basis (i) for developing a statistical model for analyzing variations in vestibular anatomy (Fritscher et al., submitted) and (ii) for realistic models of human anatomy for analyzing the effects of different implantation strategies on vestibular nerve stimulation. In order to transform these specimens and instances of the statistical model into *in silico* models used for the analysis of vestibular stimulation scenarios, an efficient computational workflow is required.

In this work a (semi-)automatic modular workflow for the stepwise transformation of segmented vestibular anatomy to a virtual model with subsequent analysis is presented, where the majority of the workflow (except the segmentation of the μCT scans) was developed based on open-source tools. Results obtained by the workflow based on human anatomy are compared with corresponding results from literature obtained by *in vivo* measurements and simulation results. While this work focuses on the development of the modular workflow and the comparison with data obtained from literature, the reader is additionally referred to the work of Schier et al. (submitted), which employs this workflow for the investigation of stimulation scenarios for human vestibular ampullary nerves.

## 2. Materials and methods

### 2.1. Workflow

**Figure 2:**
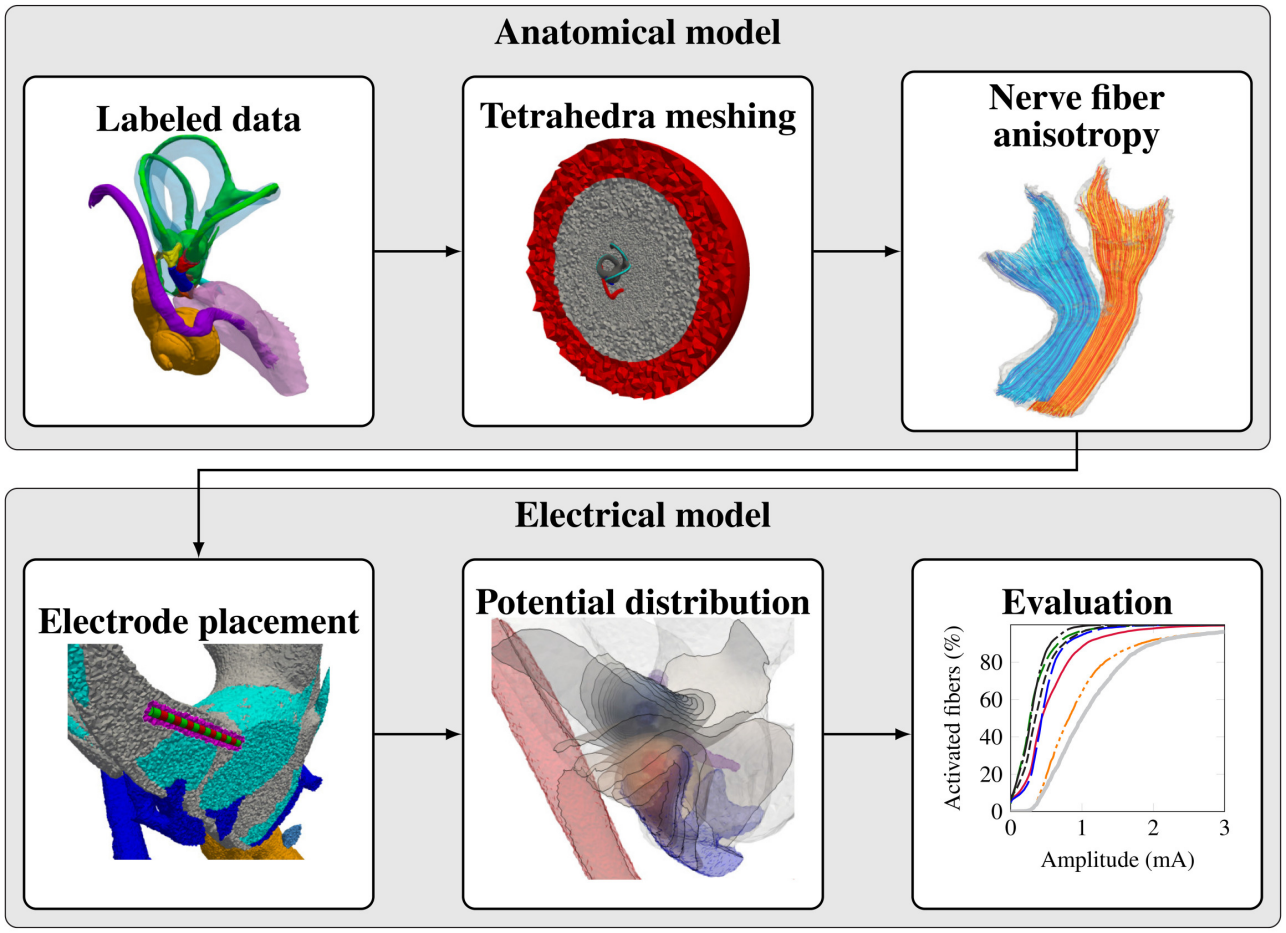
Modular workflow for evaluating stimulation scenarios on a given vestibular anatomy.

The workflow for creating a representative model of an anatomical input dataset is depicted in Figure 2. The modular components of the workflow are described in the following subsections.

#### 2.1.1. Labeled data of vestibular specimen

##### 2.1.1.1. Labeled datasets

The bodies, from which the vestibular specimens were excised, were donated to the Division of Clinical and Functional Anatomy of the Innsbruck Medical University by people who had given their informed consent for their use for scientific and educational purposes prior to death (McHanwell et al., 2008; Riederer et al., 2012).

All cadavers were preserved using an arterial injection of a formaldehyde-phenol solution/an alcohol-glycerin solution and immersion in phenolic acid in water for 1–3 months (Platzer et al., 1978).

The vestibular specimens were obtained after post-mortem by surgical excision and were then fixed in 4% paraformaldehyde. The excised specimens did not exhibit any clinical abnormalities or pathologies associated with known inner ear disorders. Following fixation in formaldehyde the specimens were washed thrice in 1 × Phosphate buffered saline (PBS) and then in freshly prepared 0.1 M Cacodylate buffer. Following which the specimens were placed in a solution of Osmium tetroxide (OsO_4_) in Cacodylate buffer for 48 h at 4°C with constant shaking. The specimens were then washed again in cacodylate buffer thrice to remove the traces of Osmium tetroxide and left in 1 × PBS. Following which the specimens were decalcified in 20% Ethylene diamine tetra acetic acid dissolved in 1 × PBS over a period of 40 days. The decalcified temporal bones were then washed in 1 × PBS several times and left in 1 × PBS with 0.01% Sodium azide and scanned again using a Zeiss XRM XRadia-35 μCT scanner at a voxel resolution of 15 μm, with scan projections set at 1,000, integration time of 1,600 ms and the current used at 45 keV.

For these datasets, structures of the inner ear were manually labeled using Amira® 6.3.0 (Thermo Scientific™, Hillsboro, Oregon, USA). Comparable approaches for 3D reconstruction have also been performed in model organisms (see for example Kopecky et al., 2012 for the segmentation of murine inner ear structures). In addition to the structures of the vestibular system (endolymph, perilymph, N. ampullaris posterior, N. ampullaris anterior, N. ampullaris lateralis, common section of the N. ampullaris anterior and N. ampullaris lateralis, N. utricularis, N. saccularis, sensory epithelia), also neighboring structures of the cochlea (N. cochlearis, scala tympani/media/vestibuli), the N. facialis and the IAC were labeled. The labeled datasets serve as input for the statistical model and the virtual models of individual datasets for the analysis of vestibular stimulation protocols.

##### 2.1.1.2. Statistical shape model

A deformation-based morphometry approach has been developed to quantitatively analyze morphological variations of single structures as well as multi-object ensembles within the vestibular system and to generate models based on the statistical shape variations that can be used as a basis for the evaluation of electrical stimulation scenarios. For a more detailed description of this model and for an overview of statistical variations in the vestibular anatomy, the reader is referred to Fritscher et al. (submitted).

Based on the manual segmentation of 31 specimen datasets of temporal bones, statistical shape models for the following structures have been created:

- Perilymph
- Endolymph
- Bony labyrinth (approximated using a combined label of endolymph and perilymph)
- N. ampullaris anterior and N. ampullaris lateralis
- N. ampullaris posterior
- N. facialis

The mean shape of the statistical model was then used as an input for the workflow to analyze defined electrode configurations (see section 2.1.4) and compare the results with simulations based on individual datasets (see section 3.2).

Since the main aim of the used statistical shape model described in Fritscher et al. (submitted) was the analysis of anatomical variations of the components described in the previous listing, the remaining components (N. utricularis, N. saccularis, cochlea, and IAC) were not considered in the statistical model and, consequently, these constituents were not available for the evaluation of vestibular stimulation scenarios using the statistical model by the described framework. However, for the analysis of vestibular stimulation scenarios *in individual datasets*, all labeled components shown in Figure 1 were considered.

#### 2.1.2. Tetrahedra meshing

The high-resolution labeled voxel data is converted into a tetrahedra mesh using the Computational Geometry Algorithms Library (CGAL) (Alliez et al., 2017; The CGAL Project, 2017) and TetGen (Si, 2015). Central components of the vestibular system (vestibular nerve branches, bony labyrinth) were meshed in a higher resolution than the surrounding regions (cochlea, IAC). Further surrounding structures (e.g., temporal bone, skin, vasculature, air inclusions) could not be labeled due to the excision and chemical preparation of the inner ear previous to μCT-imaging. The labeled components are embedded in a bone sphere (diameter: 5 cm) that is again surrounded by a saline layer (1 cm thickness) to consider the surrounding structures mainly consisting of bone in the simulation of the quasistationary potential distribution. A similar approach was also used in the models of Marianelli et al. (2015).

#### 2.1.3. Nerve fiber anisotropy and fiber generation

Nerve tissue exhibits a significantly higher electrical conductivity along the nerve fiber axis compared to the transversal directions normal to the nerve fiber axis (Hayden et al., 2011). The nerve fiber orientation is estimated separately for each nerve branch to consider the anisotropic electrical conductivity of the neural tissue in the model. The workflow for estimating the nerve fiber orientation and fiber generation is described in the following paragraphs. A prerequisite to the described approach is that the nerve volume, for which the nerve fiber orientation should be computed, is present as a single connected region.

##### 2.1.3.1. Definition of the starting surface

**Figure 3:**
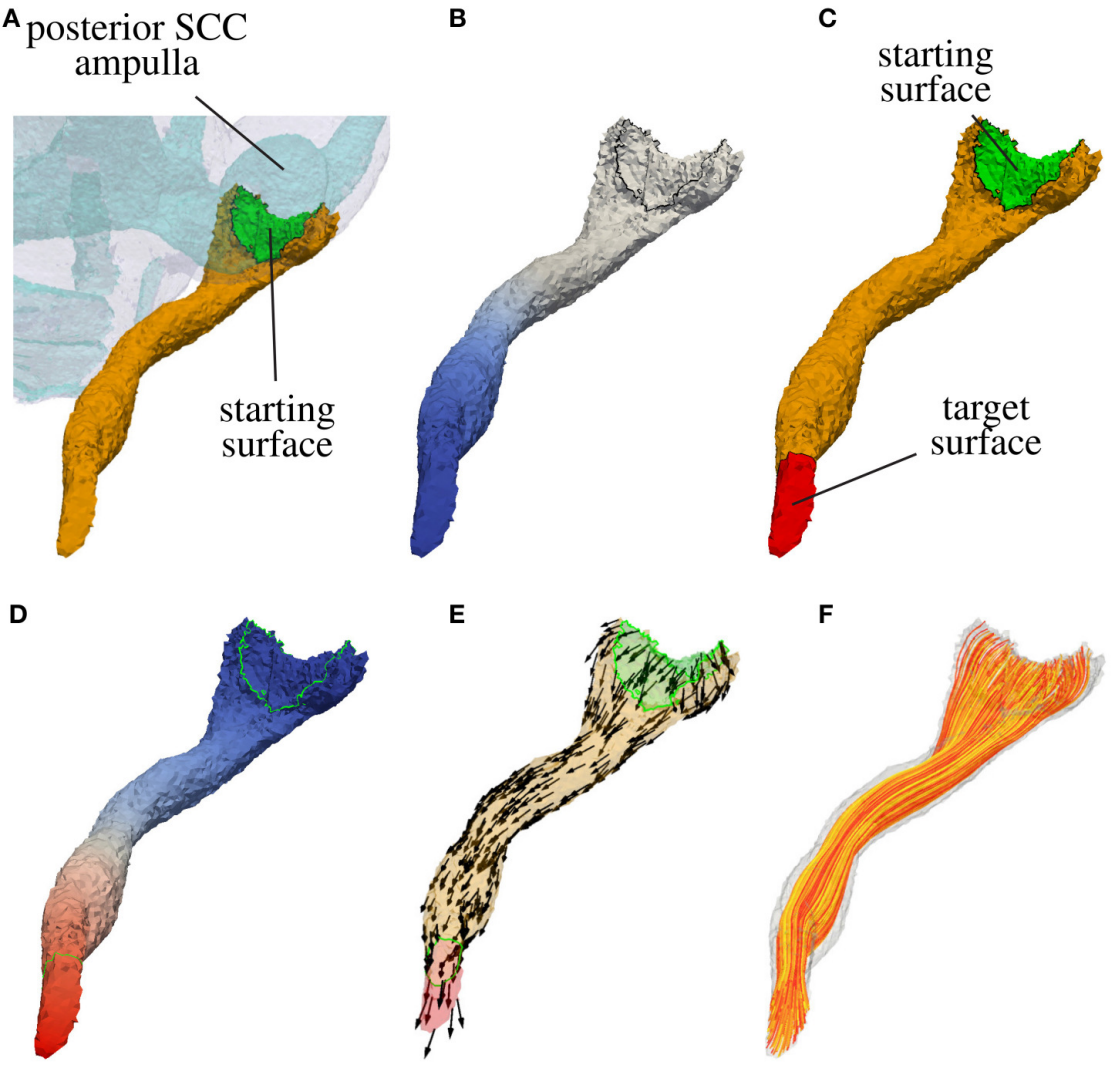
Computation of nerve fiber orientation of the N. ampullaris posterior in an exemplary dataset. **(A)** Extraction of the starting surface (common surface of nerve volume and endolymphatic space). **(B)** Computation of distance field relative to starting surface (blue color depicts lower potential in volume conductor simulation). **(C)** Highlighted starting and target surface used for the computation of the nerve fiber orientation field. **(D)** Resulting potential distribution of the volume conductor simulation after applying a low external potential ϕ_*e*_ at the starting surface (colored in blue) and a high external potential ϕ_*e*_ at the target surface (colored in red). **(E)** Normed gradient field of the potential distribution depicted in **(D)** defining the nerve fiber orientation. **(F)** Four hundred nerve fibers generated by streamlines based on the gradient field depicted in **(E)**.

In a first step the surface of the nerve volume in contact with the sensory epithelium is extracted. This surface defines the starting surface for the modeled nerve fibers passing through in the labeled nerve volume. If the sensory epithelium could not be labeled reliably, the surface connecting the nerve volume with the volume of the endolymphatic space is considered as sensory epithelium. In Figure 3A the starting surface of the N. ampullaris posterior to an exemplary dataset is depicted in green.

For the N. facialis the starting surfaces must be estimated differently, as only a central component of the N. facialis is considered in the model in close vicininty around the vestibular system and no reference volumes are available to determine the starting surface. The estimation of the starting surface for the N. facialis can be performed similarly as described for the target surface in the following step.

##### 2.1.3.2. Computation of the target surface

In a next step a target surface is computed that defines the direction of the nerve fiber growth beginning from the starting surface. To obtain the target surface, the most distant point relative to the starting surface is calculated by performing a stationary volume conductor simulation on the tetrahedra-meshed volume of the nerve Ω_*n*_ based on the *Laplace equation*

(1) $$\begin{array}{l} \Delta \phi \left(\text{x}\right)=0;\;\text{x}\in \Omega_{n}, \end{array}$$

where ϕ is a measure equivalent to a potential at position ***x***. By applying a constant potential at the starting surface Γ_*s*_

(2) $$\begin{array}{l} \phi \left(\text{x}\right)=0;\;\text{x}\in \Gamma_{s} \end{array}$$

and a constant outflow over the remainder of the surface

(3) $$\begin{array}{l} -\nabla \phi \left(\text{x}\right)\cdot {\text{n}}_{\Gamma }\left(\text{x}\right)=1;\;\text{x}\in \left\{\Gamma \backslash \Gamma_{s}\right\} \end{array}$$

with ***n***_Γ_ being the outward pointing surface normal, the surface point with the lowest potential ϕ corresponds approximately to the most distant surface point in relation to the starting surface. The target surface Γ_*t*_ is defined by applying a Dijkstra algorithm and finding all surface elements within a specific range surrounding the approximated most distant point. Figure 3B shows the distance field relative to the starting surface and in Figure 3C the target surface of the N. ampullaris posterior to an exemplary dataset is depicted in red.

For the N. ampullaris anterior and N. ampullaris lateralis, a common volume is considered for the determination of the starting surfaces and a shared target surface is calculated for both branches.

If the N. facialis is shaped as a tube like structure with rounded endings, the starting surface can be determined in a similar way to the computation of the target surface. Initially, a random surface element of the N. facialis is selected and defined as a temporary starting surface Γ_*s*_. After applying the boundary conditions (Equations 2 and 3), Equation (1) is solved, and similarly to the creation of the target surface, the starting surface is defined by all surface elements within a specific distance relative to the point with the lowest potential in the resulting potential distribution. Based on the starting surface, the target surface is computed analogously to the target surfaces of the vestibular nerves.

If an automatic determination of the starting and target surfaces with the proposed approach is not possible (e.g., no labeled sensory epithelium/endolymphatic space available, no direct contact of vestibular nerve tissue with sensory epithelium/endolymphatic space, rough starting/target surfaces due to excision of the vestibular system previous to μCT imaging) manually defined starting and target surfaces are used in the next steps.

##### 2.1.3.3. Computation of the fiber orientation field

Based on the defined starting surface Γ_*s*_ and target surface Γ_*t*_ the nerve fiber orientation field is calculated based on an additional volume conductor simulation as described in the previous section 2.1.3.2 with adapted boundary conditions: A *Cauchy boundary condition*

(4) $$\begin{array}{l} -\nabla \phi \left(\text{x}\right)\cdot {\text{n}}_{\Gamma }\left(\text{x}\right)=\alpha \left(\text{x}\right)\left[\phi \left(\text{x}\right)-\phi_{e}\left(\text{x}\right)\right];\;\text{x}\in \Gamma \end{array}$$

is applied on the whole surface of the nerve volume, where ϕ_*e*_ defines a location dependent external potential and the location dependent coefficient α (m-2) scales the effect of the externally applied potential ϕ_*e*_ on the internal potential ϕ. By defining

(5) $$\begin{array}{l} \phi_{e}\left(\text{x}\right)=\left\{ \begin{array}{cc} 1;\; & \text{x}\in \Gamma_{t}\\ 0;\; & \text{x}\in \left\{\Gamma \backslash \Gamma_{t}\right\}\\ \; \end{array}\right. \end{array}$$

and

(6) $$\begin{array}{l} \alpha \left(\text{x}\right)=\left\{ \begin{array}{cc} \alpha_{s};\; & \text{x}\in \Gamma_{s}\\ \alpha_{t};\; & \text{x}\in \Gamma_{t}\\ 0;\; & \text{x}\in \left\{\Gamma \backslash \left(\Gamma_{s}\cup \Gamma_{t}\right)\right\} \end{array}\right. \end{array}$$

with α_*s*_, α_*t*_ > 0, a potential difference between the starting and target surface is applied with an insulating boundary at the rest of the surface. After solving Equation (1) under consideration of the described boundary condition, the potential ϕ raises continuously from the starting surface Γ_*s*_ to the target surface Γ_*t*_ (see Figure 3D). The vector field estimating the nerve fiber orientation ***F*** can then be computed by evaluating the normed gradient of the potential distribution ϕ:

(7) $$\begin{array}{l} \text{F}\left(\text{x}\right)=\frac{\nabla \phi \left(\text{x}\right)}{\left|\nabla \phi \left(\text{x}\right)\right|} \end{array}$$

The adjustment of α_*s*_ and α_*t*_ allows for a “fine-tuning” of the nerve fiber orientation at the starting and target surface, where larger values for α_*s*_ and α_*t*_ lead to a steeper entrance/exit angle of the nerve fiber orientation at the starting and target surfaces, respectively. Figure 3E depicts the resulting vector field ***F*** defining the nerve fiber orientation for the N. ampullaris posterior to an exemplary dataset with α_*s*_ = α_*t*_ = 100. ***F*** is consequently used (i) to consider the anisotropic electrical conductivity of neural tissue in the computation of quasistationary potential distributions in the inner ear and (ii) to generate realistically distributed and running nerve fibers to calculate the excitation in targeted and non-targeted nerve branches to evaluate the selectivity of investigated stimulation scenarios (Hayden et al., 2011; Schier et al., submitted).

##### 2.1.3.4. Nerve fiber generation

Based on the computed nerve fiber orientation field ***F*** realistical nerve fibers are generated. It is necessary to reproduce the nerve fibers in the model as realistically as possible to ensure accurate neural responses. The nerve fiber generation is independent from nerve morphology (e.g., thickness, twisting, bifurcation) and produces a set of artificial neuron trajectories through each nerve volume.

Each artificial neuron grows from the starting surface(s) to the target surface of their respective nerve volume. The origins of the neurons are randomly scattered points all over the starting surfaces. Beginning from those points a stream tracing algorithm by the C++ library VTK (Schroeder et al., 2006) is used to generate the neural trajectories along the anisotropy vector field (see Figure 3F). The algorithm terminates when a trajectory leaves the nerve volume. Only if the tracing of a trajectory terminates by crossing the target surface, it is accepted into the fiber set used for the simulation of neural excitation. Otherwise, a different start point is chosen until the termination criterion is met and the desired number of artificial neurons is bundled in the fiber set.

Four hundred neurons were generated for the N. ampullaris anterior, N. ampullaris lateralis and N. ampullaris posterior, N. utricularis and N. saccularis as well as the N. facialis and the IAC, respectively. By considering artificially placed nerve fibers in the IAC, the stimulation of nerve fibers in the IAC is always regarded as activation of non target nerve fibers. This attributes to the fact that the exact pathways of the nerve branches within the IAC are not known in the labeled dataset and that nerve branches are slightly rotating along the IAC (Sando et al., 1972), making a direct assignment of nerve fibers to specific target and non target nerve branches unfeasible.

Three different neuron fiber types were considered in the model with characteristic morphologies and excitability like described previously by Hayden et al. (2011): *Irregular fibers* innervate mainly the central part of the sensory epithelia, while *regular fibers* are mainly found in the peripheral area. *Dimorphic fibers* innervate all regions of the sensory epithelia. Each generated fiber is assigned to one described type depending on the posiiton of the starting position on the sensory epithelia. For a more detailed description of the applied neuron model the reader is referred to Schier et al. (submitted), who adapted the neuron model described in Hayden et al. (2011) to consider human vestibular nerve fiber morphology.

Based on the generated neuronal pathways, nodes of Ranvier are defined for myelinated neurons along the nerve fiber paths considering neuron type dependent internodal distances (Schier et al., 2016; Schier et al., submitted). The nodes of Ranvier define the interface between the simulated potential distributions generated by the defined electrodes (see section 2.1.5) and the neuron model (see section 2.1.6).

#### 2.1.4. Electrode placement

Elementary electrode designs such as spherical electrodes and electrode arrays are used to analyze the effects of exact electrode positionings and electrode spacings in mono- and multipolar stimulation scenarios. Also realistic electrode designs can be considered in the model to test the effects of insulating materials on potential distributions and the stimulation outcome (McIntyre and Grill, 2001).

**Figure 4:**
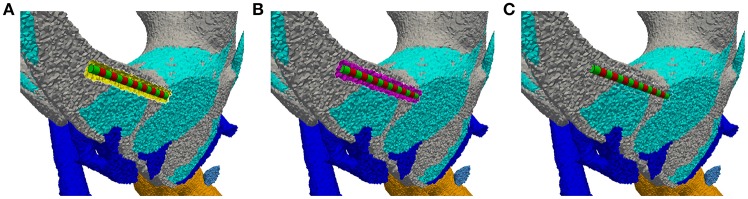
Insertion of an electrode array into the intra-labyrinthine space depicted on a cross section of the vestibular system. **(A)** Created cavity at planned electrode location (yellow surface elements) with inserted electrode array (green/red regions). **(B)** Gap filled with tetrahedra elements (purple region). **(C)** Originally present material properties applied to the filling region.

The electrodes are inserted into the previously defined anatomical model by (i) removing all tetrahedra within a defined distance around the target position of the electrode, (ii) inserting the tetrahedra meshed geometry of the electrode into the created gap (see Figure 4A), (iii) filling the gap with a fitted tetrahedra mesh (see Figure 4B) and (iv) assigning the original region properties to the elements filled into the gap (see Figure 4C). To compare different non-overlapping electrode configurations without the need to adapt the mesh, multiple electrodes can be inserted within one anatomical model. If only a subset of the inserted electrodes is needed in a simulation scenario, the originally present region properties of the anatomical model are assigned to all other electrodes.

#### 2.1.5. Potential distribution

An effective approach described by Hayden et al. (2011) was applied to calculate the temporal progress of the potential distribution in the modeled volume of the inner ear during different stimulus forms: All tissues in the model were considered purely conductive without reactance due to shorter dielectric relaxation times of biological tissues with respect to the time scale of the applied stimulus pulses (Spelman et al., 1982), allowing to assume quasistatic potential distributions at discrete timestamps during stimulation. The potential distribution present during a time instance of the stimulus can therefore be determined time independently by scaling the potential distribution induced by a reference current through the active electrodes with the amplitude of the stimulus at the examined time instance.

The potential distribution of an applied unit current was calculated using the *Poisson equation* (Joucla and Yvert, 2012; Marianelli et al., 2015)

(8) $$\begin{array}{l} -\nabla \cdot \left(\sigma \left(\text{x}\right)\nabla \phi \left(\text{x}\right)\right)=\nabla \cdot \text{J}\left(\text{x}\right)=I_{s}\left(\text{x}\right), \end{array}$$

where **σ** defines the *electrical conductivity* (S m-1), ϕ is the *potential* (V), ***J*** is the *current density* (A m-2) and *I*_*s*_ is the *current source density* (A m-3) at location ***x***. The electrical conductivity values used in the simulations are listed in Table 1.

**Table 1:** Electrical conductivity values used in the computation of potential distributions.

**Tissue/material**	**σ (S m^−1^)**
Bone	0.0139^a^
Nerve longitudinal	0.3333^a^
Nerve transversal	0.0143^a^
Cochlear nerve	0.1738
Scala tympani/media/vestibuli	2.0^a^
Endolymph/Perilymph	2.0^a^
Saline layer	2.0^b^
Electrode	1.0 × 10^6^

Values were obtained from

a Hayden et al. (2011) (vestibular system) and

b *Marianelli et al. (2015) (saline layer surrounding the bone sphere). The average of the longitudinal and transversal conductivity of nerve tissue was considered for the cochlear nerve as no nerve fibers were generated for this nerve*.

The unit current injected by the active electrodes was considered by distributing it uniformly over the regions covered by the active electrode(s) Ω_*a*_

(9) $$\begin{array}{l} I_{s}\left(\text{x}\right)=\frac{1}{V_{a}};\;\text{x}\in \Omega_{a} \end{array}$$

where *V*_*a*_ is the *volume of the active electrode(s)* (m3). In bipolar stimulation scenarios (both active and closely located reference electrodes present in the vestibular system) a *constant reference potential* of 0 V was applied at regions covered by the reference electrode Ω_*r*_

(10) $$\begin{array}{l} \phi \left(\text{x}\right)=0;\;\text{x}\in \Omega_{r} \end{array}$$

and a *homogeneous Neumann boundary condition*

(11) $$\begin{array}{l} -\left(\sigma \left(\text{x}\right)\nabla \phi \left(\text{x}\right)\right)\cdot {\text{n}}_{\Gamma }\left(\text{x}\right)=0;\;\text{x}\in \Gamma_{\text{sal}} \end{array}$$

was defined at the outer surface of the saline layer Γ_sal_. In monopolar stimulation scenarios (active electrode located in the vestibular system, distant reference outside the modeled domain), the distant reference electrode was considered in the model by applying a constant potential at the outer surface of the saline layer Γ_sal_ (Marianelli et al., 2015):

(12) $$\begin{array}{l} \phi \left(\text{x}\right)=0;\;\text{x}\in \Gamma_{\text{sal}} \end{array}$$

#### 2.1.6. Evaluation

Based on the described model, specific key figures are determined for tested stimulation protocols, describing the selectivity of excitation for targeted nerve fibers. In addition, injected charge and energy expenditure are evaluated to obtain a measure for tissue damage and battery life, respectively (Hayden et al., 2011; Schier et al., 2016; Schier et al., submitted).

In this section, only the main concepts and algorithms used for selectivity analysis of stimulation scenarios are described, as these measures are used in the evaluations summarized in this work. The reader is referred to Schier et al. (submitted) for more detailed information about the neuronal models, the described measures and further key figures that were used for the evaluation of additional electrode configurations and stimulus waveforms.

##### 2.1.6.1. Nerve fiber stimulation

For the determination of nerve fiber stimulation, realistic neuron models are applied individually to all generated nerve fibers and the potential distributions generated by stimuli applied to the inserted electrodes are interpolated at the nodes of Ranvier to define the extracellular potential input for the neuron models (Hayden et al., 2011; Marianelli et al., 2015; Schier et al., 2016; Schier et al., submitted). Hayden et al. (2011) described a well suited axon model especially fitted for vestibular neurons and used it to analyze the effect of different electrode configurations and pulse waveforms on the selective stimulation of vestibular afferents of chinchillas. For the N. facialis the model described by Frijns et al. (1994) was used. The neuronal models were adapted to consider morphological parameters of human nerve fibers, and also afterhyperpolarization effects were adapted correspondingly.

The current amplification factor threshold required for activating the neuron using a defined pulse waveform is computed for each neuron present in the model. Based on these thresholds, the ratio of activated neurons per nerve branch (fiber recruitment) is brought in relation to the amplification factor of the stimulus waveform. This comparison enables the determination of the optimal stimulation current for selective nerve stimulation (Hayden et al., 2011).

##### 2.1.6.2. Area under the ROC curve

A key figure for the selectivity of a specific electrode configuration and stimulus waveform was introduced based on a statistical measure used for the evaluation of classifiers (Schier et al., 2016; Schier et al., submitted): The *receiver operating curve (ROC)* is determined for the selected stimulation scenarios (protocols) by plotting the fiber recruitment of the target nerve (true positive rate) against the fiber recruitment of the maximally activated non-target nerve (false positive rate) over a continuously increasing stimulus amplitude to determine a key figure for the selectivity of a specific electrode configuration and stimulus waveform. By computing the *area under the ROC curve (AUC)*, a selectivity measure in the range between 0 (all nerve fibers of a non-target nerve are activated while no nerve fiber of the target nerve is activated) and 1 (all nerve fibers of the target nerve are activated while no nerve fiber of any non-target nerve is activated) is obtained, where higher values of the AUC represent a higher selectivity.

### 2.2. Analyzed vestibular stimulation scenarios

Labeled datasets of four human vestibular systems were preprocessed and analyzed using the presented workflow to verify the computational framework and to analyze the efficacy of defined stimulation protocols using different anatomies. The relevant data of the individuals, from which the vestibular specimens were obtained, are summarized in Table 2. The temporal bone specimens belonged to healthy individuals with an average age of 72 and each of these specimens had an average post mortem time of 7 h before they were excised.

**Table 2:** Data of individuals, from which the individual virtual models of the vestibular system were created.

**ID**	**Gender**	**Age**	**Post mortem time (h)**
1	Male	85	6
2	Male	54	6
3	Male	78	10
4	Male	70	6

*The ID of the specimen corresponds to the model number in Table 3*.

In addition, the mean shape of the described statistical model of the vestibular system was generated and used as an input for the described workflow. Spherical virtual electrodes (diameter: 200 μm) were positioned based on the orientation of targeted ampullary nerve branches and their corresponding semicircular canals. Four electrode configurations per ampullary nerve branch were analyzed (see **Figure 7C**):

For the monopolar configuration an electrode was positioned in a distance of 750 μm from the sensory epithelium along the transversal axis of the distalmost portion of the target ampullary nerve. Three bipolar configurations with an electrode distance of 1 mm were positioned parallel to the following axes: An *axial dipole* electrode pair was positioned tangential to the SCC (active electrode more distant/reference electrode closer to the vestibule). A *transverse parallel dipole* was oriented parallel to the transverse axis of the distalmost portion of the target nerve (active electrode closer/reference electrode more distant to the sensory epithelium). A *transverse perpendicular dipole* electrode configuration was positioned with a dipole moment perpendicular to the *axial dipole* and the *transverse parallel dipole* (active electrode left/reference electrode right in the view from the SCC into the vestibule with the sensory epithelium of the target nerve below the monopolar electrode position). The center position of the bipolar electrode configurations conformed to the monopolar electrode position.

Fiber recruitment curves, AUCs and stimulus amplitudes required to stimulate 80% of the target nerve fibers were evaluated using a cathodic-first symmetric biphasic stimulus waveform with a phase duration of 200 μs and an interphase gap of 30 μs (see **Figure 8M**). The described electrode configurations and stimulus waveform were chosen to allow for a comparison with results obtained by a virtual model described by Hayden et al. (2011), who used equivalent stimulation scenarios.

## 3. Results

### 3.1. Nerve fiber anisotropy and fiber generation

**Figure 5:**
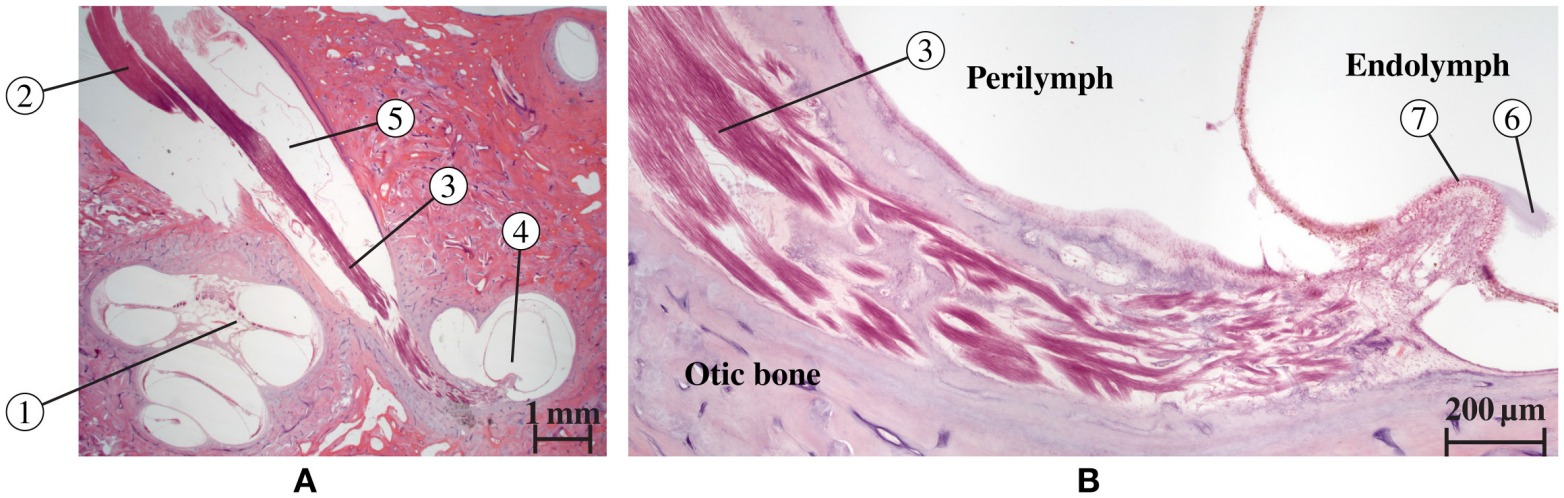
Histological section of a human inner ear (Hematoxilin-Eosin staining) along the N. ampullaris posterior. **(A)** Cochlea (1) and nerves [N. cochlearis (2) and N. ampullaris posterior (3)] traveling through the IAC. The N. ampullaris posterior innervates the posterior ampullar crest (4). The inferior vestibular ganglion (5) is visible and contains the bipolar vestibular neuron somata. **(B)** Magnified view of the N. ampullaris posterior (3) heading toward the sensory organ displaying the parallel organization of nerve fiber bundles. The cupula structure (6) is still attached to the sensory epithelium (7) within the endolymphatic compartment.

**Figure 6:**
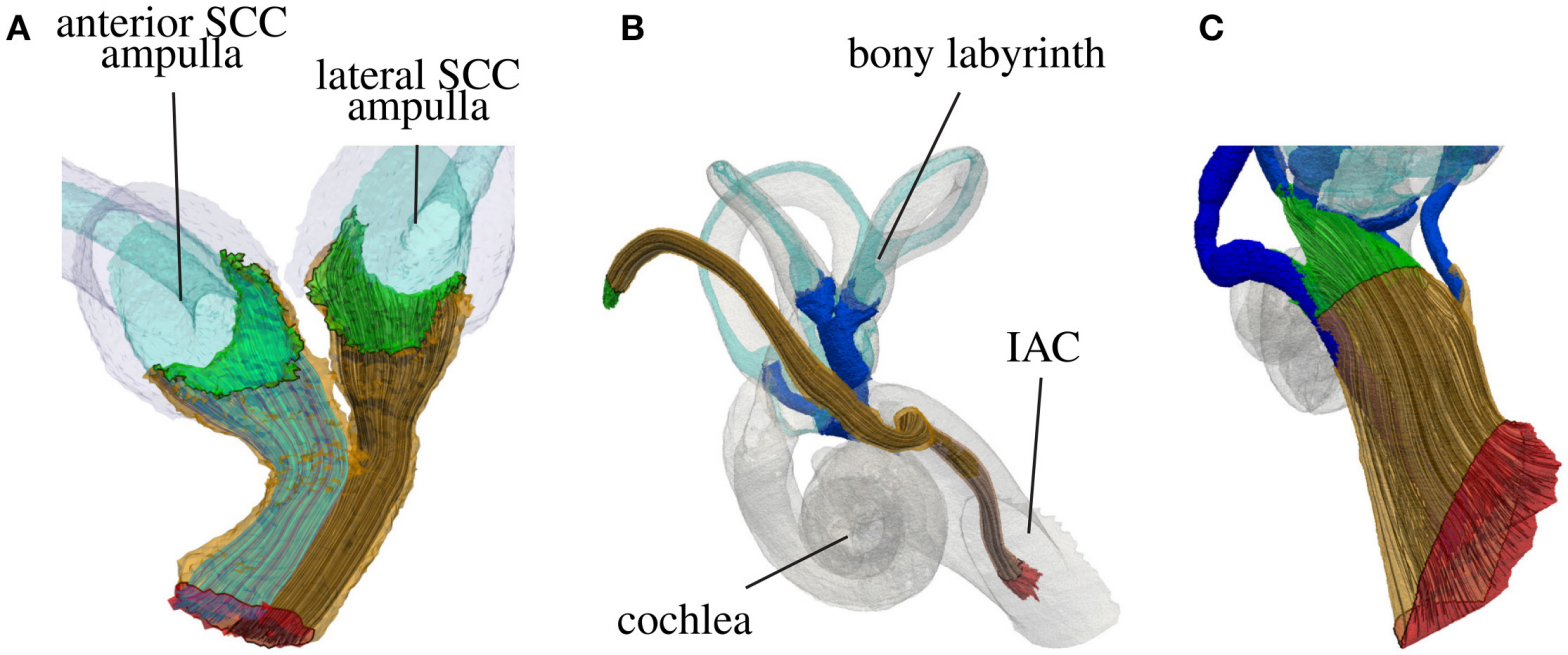
Highlighted starting surfaces (green) and target surfaces (red) and generated nerve fibers of the common nerve branch of the N. ampullaris anterior and N. ampullaris lateralis **(A)**, the N. facialis **(B)** and the IAC **(C)**.

Figure 5 shows a histological section of a human inner ear with a focus on the N. ampullaris posterior, in which the parallel organization of the nerve fibers is displayed. The neurons follow a common path starting from the sensory epithelium and running toward the IAC. Figure 6 summarizes the results of the nerve fiber anisotropy and fiber generation used (i) to consider the anisotropic electrical conductivity in the simulation of potential distributions and (ii) to evaluate the selective stimulation of single nerve branches for the common branch of the N. ampullaris anterior and N. ampullaris lateralis, the N. facialis and the IAC. Corresponding results for the N. ampullaris posterior were already presented in Figure 3. For the nerve branches innervating the utricle and the saccule the anisotropy vector field and the nerve fibers are computed equivalently as described for the N. ampullaris posterior and are therefore not separately depicted. In this exemplary dataset, the starting and target surfaces of the N. facialis and the IAC were manually defined. Generated fibers of the ampullary nerves and the nerves innervating the otolithic organs start evenly distributed over the sensory epithelium and narrow depending on the cross-sectional area of their respective foramina while maintaining cross-sectional positions relative to neighboring nerve fibers.

### 3.2. Evaluation of stimulation protocols on individual and statistical datasets

#### 3.2.1. Evaluation using the mean shape of the statistical model

**Figure 7:**
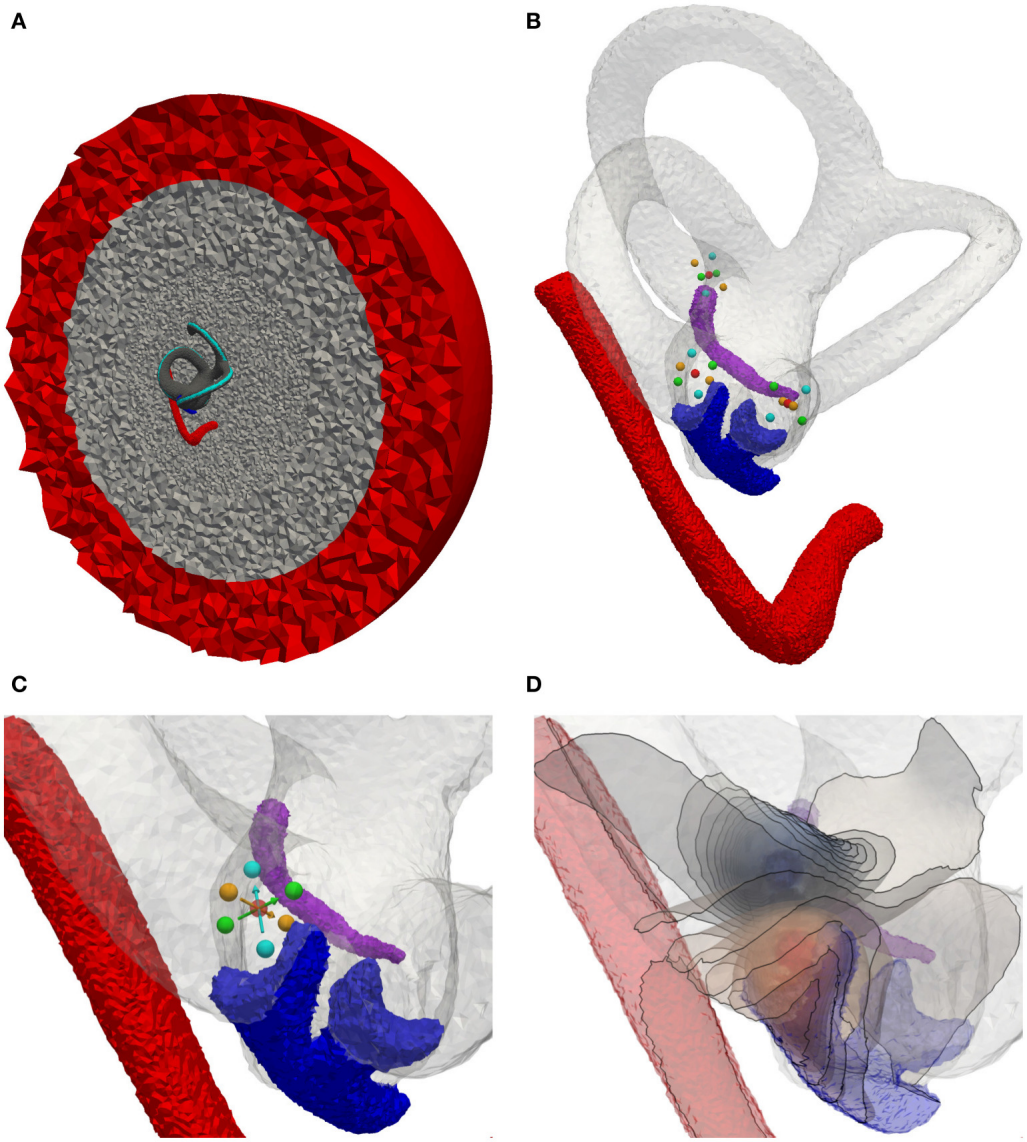
Electrode positioning in the mean shape model. **(A)** Model with cross section of surrounded bone (gray) and saline layer (red). **(B)** Model with inserted electrodes based on the definition of Hayden et al. (2011). **(C)** Focus on the ampulla of the lateral SCC. Red sphere, Electrode used for monopolar stimulation on the surface of the surrounding saline layer. Cyan spheres, Transversal parallel electrode configuration. Green spheres, Transversal perpendicular electrode configuration. Orange spheres, Axial electrode configuration. Arrows point from active electrode to reference electrode. **(D)** Exemplary visualization of isopotentials of transverse parallel electrode configuration.

Figure 7 depicts the generated tetrahedra mesh of the statistical model with inserted spherical electrodes and an exemplary potential distribution (illustrated by isopotentials) of a transverse parallel electrode configuration close to the ampulla of the anterior SCC. Based on this model, the described electrode configurations were simulated and evaluated using fiber recruitment curves and the AUC as described in section 2.1.6.

**Figure 8:**
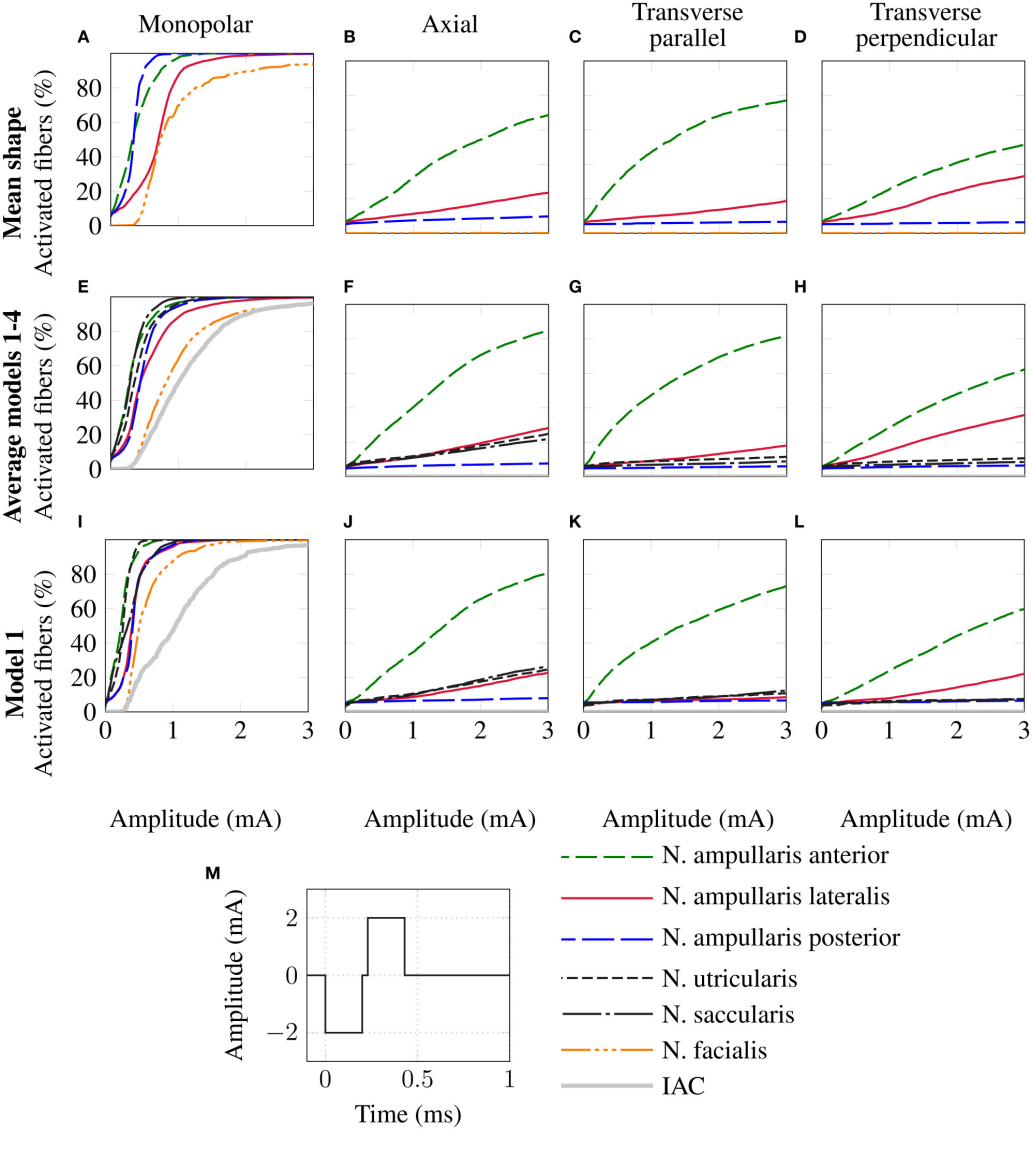
Comparison of fiber recruitment curves of the mean shape of the statistical model **(A–D)**, the averaged fiber recruitment curves of four manually segmented datasets **(E–H)** and of one individual dataset (Model 1 from Table 3) **(I–L)** for monopolar **(A,E,I)**, axial **(B,F,J)**, transversal parallel **(C,G,K)** and transversal perpendicular **(D,H,L)** electrode configurations positioned close to the anterior ampullary nerve using a cathodic-first symmetric bipolar stimulus waveform **(M)**.

The top row of Figure 8 shows the fiber recruitment curves of the mean shape of the statistical model using a cathodic-first symmetric bipolar stimulus waveform with a phase duration of 200 μs and a phase gap of 30 μs of electrode configurations close to the ampulla of the anterior SCC. The AUC values and the stimulus amplitude required to activate 80% of the target nerve are summarized in the *Mean model* column in Table 3 for all ampullary nerve branches. The monopolar electrode configuration showed greater electrode-nerve coupling (low stimulus amplitude required for stimulating the target nerve) and a lower selectivity compared to the bipolar protocols. All vestibular nerve branches as well as the N. facialis were activated within a small range of the stimulus amplitude. The transversal parallel electrode configuration showed the highest selectivity over all electrode configurations in the mean shape of the statistical model. For all bipolar electrode configurations positioned in the ampulla of the anterior SCC, the non-target nerve with the highest fiber recruitment is the N. ampullaris lateralis, followed by the N. ampullaris posterior, in which fibers were only active due to effects induced by spontaneous discharge regularity considered in the neuron model, which is characteristic for vestibular nerves (Fernandez et al., 1988). The N. facialis was only activated in the monopolar electrode configuration. Similar results were obtained in the simulations with other targeted ampullary nerves.

**Table 3:** AUC values and stimulus amplitude required to stimulate 80% of the target nerve for electrode configurations (El. conf.) in proximity to the specified target nerves (Target) for the mean shape of the statistical model (Mean model) and four manually segmented datasets (Models 1–4).

**Target**	**El. conf**.	**AUC/stimulus amplitude for 80% target nerve activation (mA)**				
		**Mean model**	**Model 1**	**Model 2**	**Model 3**	**Model 4**
Anterior	Monopolar	0.485/0.575	0.535/0.334	0.553/0.341	0.356/0.819	0.386/0.482
	Axial	0.791/4.285	0.793/2.955	0.807/2.400	0.839/2.604	0.772/2.420
	Trans. par.	**0.851/3.521**	**0.882/3.605**	**0.826/2.512**	**0.862/2.762**	**0.835/2.342**
	Trans. perp.	0.609/7.668	0.748/6.004	0.608/5.172	0.613/5.215	0.690/3.982
Lateral	Monopolar	0.459/0.571	0.443/0.407	0.482/0.380	0.403/0.780	0.358/0.601
	Axial	0.795/3.572	0.753/3.435	0.815/3.053	**0.811/3.601**	0.774/3.145
	Trans. par.	**0.887/2.266**	**0.869/2.789**	**0.835/2.393**	0.779/4.138	**0.865/2.393**
	Trans. perp.	0.607/7.414	0.836/8.102	0.742/3.061	0.613/12.859	0.677/7.324
Posterior	Monopolar	0.891/0.261	0.638/0.302	0.642/0.302	0.499/0.464	0.459/0.444
	Axial	0.915/2.980	0.814/3.270	0.831/2.293	0.807/3.876	0.845/2.494
	Trans. par.	**0.920/3.291**	0.901/3.484	0.872/3.256	**0.905/4.012**	**0.885/2.279**
	Trans. perp.	0.919/7.281	**0.902/8.461**	**0.884/5.594**	0.871/7.865	0.875/6.523

*Most selective electrode configuration for a particular nerve and model is highlighted in bold font*.

#### 3.2.2. Evaluation using individual datasets

The results of the simulation of the monopolar and bipolar electrode configurations positioned close to the ampulla of the anterior SCC based on the labeled datasets of the four human vestibular systems using a symmetric bipolar stimulus waveform are summarized in the second row of Figure 8. The corresponding AUCs and the stimulus amplitude required to activate 80% of the target nerve are listed in Table 3 (Models 1–4).

Monopolar electrode configurations showed great electrode nerve coupling at a cost of the worst selectivity compared to bipolar electrode configurations, correspondingly to the results described for the mean shape of the statistical model. The transverse parallel electrode configuration showed the highest AUC values for the majority of the analyzed scenarios. Additionally to the nerves considered in the simulation of the mean shape, the results for the individual datasets also consider the stimulation of nerve branches innervating the otolith organs and the inner auditory canal. The N. utricularis and N. saccularis show a high sensitivity to monopolar stimulation scenarios. In the displayed stimulus amplitude range between 0 mA and 3 mA, the N. facialis and the IAC showed only activation in monopolar electrode configurations. For all models and target nerves, the transverse perpendicular electrode configuration showed the worst electrode nerve coupling (highest stimulus amplitude required to stimulate 80% of the target nerve). The results of the individual datasets show a significantly lower AUC in the posterior monopolar electrode configuration compared to the results obtained by the mean shape of the statistical model.

The third row of Figure 8 shows the fiber recruitment curves for one individual dataset (Model 1 in Table 3). Similar results for the fiber recruitment were obtained for the individual model when compared to the averaged fiber recruitment curves of the four datasets (Models 1–4) in the second row of Figure 8. A higher sensitivity of the N. ampullaris lateralis, the N. facialis and the N. utricularis was observed in the monopolar electrode configuration of the individual model (see Figure 8I) compared to the averaged results of the four datasets (see Figure 8E). On the other hand, a lower sensitivity was obtained for the N. saccularis in the individual model, which showed the higest sensitivity in the averaged fiber recruitment curves of the four datasets. In the axial and transverse parallel electrode configurations, the N. saccularis and N. utricularis showed a higher percentage of activated neurons than the N. ampullaris lateralis when applying higher stimulus amplitudes in model 1 (see Figures 8J,K). Conversely, a higher percentage of fibers was activated in the N. ampullaris lateralis compared to the N. saccularis and N. utricularis in the averaged fiber recruitment curves of the four datasets (see Figures 8F,G).

#### 3.2.3. Comparison with data from literature

Equivalent electrode configurations and the stimulus waveform described in section 2.2 were also used by Hayden et al. (2011) to analyze selective vestibular nerve stimulation in chinchillas both *in silico* and *in vivo*. Both the fiber recruitment curves of the mean model and the individual datasets show a high qualitative similarity with their results, in which also the monopolar electrode configurations exhibited the lowest threshold for activating the target nerve as well as the poorest selectivity. The transverse parallel electrode configuration showed the best selectivity of all bipolar electrode configurations in their work. Compared to their work, a significantly higher stimulus amplitude is required to obtain a corresponding percentage of activated nerve fibers, in particular when comparing bipolar electrode configurations.

## 4. Discussion

### 4.1. Workflow

In this work, a newly developed framework for analyzing the efficacy of vestibular implants is described, which was tested based on the mean shape of a statistical model based on 31 human datasets as well as four manually labeled single datasets of human specimen. Based on the anatomical models, results presented in literature (Hayden et al., 2011) were successfully reproduced, verifying the applicability of the described framework for the evaluation of specific electrode configurations and stimulation scenarios. In the best knowledge of the authors, no detailed description of a workflow for the evaluation of vestibular implant solutions starting from the labeled anatomy was presented in literature yet. Specific modules of this workflow are based on previously described tools and methods, which are also focusing on vestibular implant optimization, which were extended to allow for a (semi-)automatic preprocessing (meshing, electrode insertion, fiber generation), simulation (computation of potential distributions and fiber activation thresholds) and analysis (computation of AUC, required current for 80% target nerve activation and energy consumption; Schier et al., submitted). The modular structure of the workflow allows for a continuous adaptation and improvement of each step in the pipeline to modify the framework to various scientific questions. While the workflow can be used for the analysis of vestibular implant solutions for a group of patients using generated instances of the statistical model as input of the workflow, also patient specific analyses of vestibular implant configurations can be performed efficiently in advance prior to a surgical procedure. The described workflow can also be applied directly on labeled inner ear anatomies of animals. In addition to the possibility to further verify the described workflow in combination with corresponding *in vivo* experiments, this also allows for testing the functional outcome of vestibular nerve branch stimulation on anatomical variations in various vestibular mutants that are readily available as for example for mice (Kopecky et al., 2012).

The segmented anatomy was embedded into a bone sphere surrounded by a saline layer to incorporate the effect of the anatomy surrounding the vestibular system on the potential distributions generated by the tested electrode configurations. Although similar approaches were considered in other models simulating potential distributions generated by vestibular implants (Marianelli et al., 2015) and other target regions (Schiefer et al., 2008; Raspopovic et al., 2011) it is expected that the consideration of realistic surroundings of the vestibular system (e.g., dura mater with high resistivity in medial direction, fluid/air inclusions in mastoid cells and the eustachian tube) in the virtual model could lead to deviations in the resulting potential distribution and, consequently, in the fiber recruitment curves, especially when using distant reference electrodes in monopolar stimulation scenarios. In our future work it is planned to integrate the generated vestibular models based on high resolution scans of human probes using μCT into more realistic surroundings obtained by CT/MRI scans.

In the simulations, all vestibular nerves and the N. facialis were considered if available in the corresponding labeled dataset. The IAC was introduced as a separate component in order to consider the cochlear nerve as a non-target nerve of vestibular stimulation in the model. Although the IAC also contains the proximal regions of the vestibular nerves (including also nerve fibers of the targeted nerve branch), the definition of the IAC as purely non-target nerve is justified, as the exact distribution of the nerve fibers within this region is not known exactly, also due to rotation of the vestibular nerve fibers within the IAC Sando et al. (1972). By this definition, the worst-case scenario for the IAC is taken into account in the evaluation of different stimulation scenarios.

A new method is presented in this work to consider realistic nerve fiber distributions and anisotropical electrical conductivities in the neural tissue, which computes a nerve fiber orientation field based on (semi-)automatically defined start and target surfaces of the respective nerves. While the (semi-)automatic definition of the start- and target surfaces needs attention from the user (automatically generated surfaces must still be checked by the user and redefined manually if necessary), the computation of the fiber orientation field based on the defined surfaces worked seemlesly for our tested datasets. Alternative approaches for the creation of nerve fibers in the vestibular system were already previously described in literature (Hayden, 2007; Hayden et al., 2011; Marianelli et al., 2015). While these alternative approaches also generate accurate nerve fibers for the evaluation of nerve branch activation, the method presented in this work allows for an efficient computation of nerve fibers and anisotropy vector fields based on only a small input of the user. In addition, the proposed algorithm is also able to generate nerve fiber orientation fields for various nerve volume shapes present in the vestibular model (like the IAC). It should also be noted that no fibers of the vestibular efferent system were considered in our model, since its distinct functional role in motor and vestibular coordination is not clear yet and varies significantly between species (Marianelli et al., 2015; Mathews et al., 2017).

The presented potential distributions were calculated based on the assumption of a negligible reactive component of impedance, analogously as assumed in other frameworks described in literature evaluating the efficiency of vestibular implants (Hayden et al., 2011; Marianelli et al., 2015). This assumption is justified by much shorter dielectric relaxation times in biological tissues than the time scale of the applied stimuli and a low percentage of spectral energy of the tested stimulus waveform above the critical threshold frequency (Spelman et al., 1982; Hayden et al., 2011). As it was shown in other studies (Davidovics et al., 2011) and by results obtained by our group (Schier et al., 2016; Schier et al., submitted), a higher selectivity and a lower energy consumption is obtained by applying shorter pulse durations. Extended computer models considering the reactive component of impedance in the tissues of the vestibular system could give a closer insight on effects of shorter pulse durations on the stimulation outcome of vestibular implants.

The AUC was used for the evaluation of selectivity of analyzed electrode configurations and stimulus waveforms (Schier et al., 2016; Schier et al., submitted). An alternative approach was already previously described in literature, which is defined by the difference between the target nerve recruitment and the averaged non-target nerve recruitment for a given stimulation scenario and stimulus amplitude (Schiefer et al., 2010; Raspopovic et al., 2011; Marianelli et al., 2015). A representative value for the assessment of the scenario can be computed by maximizing this selectivity index over the applied stimulus amplitude. Although this approach also describes a reasonable method for evaluating stimulation scenarios, we decided to use the AUC for our evaluations, as the resulting figure is independent from the applied stimulus amplitude and stricter results are obtained by using only the highest fiber recruitment as a representative of all non-target nerves.

### 4.2. Evaluation of stimulation protocols and comparison with data from literature

The described workflow was used to analyze the stimulation outcome in mono- and bipolar electrode configurations in both patient specific vestibular anatomy and the mean shape of a statistical model comprising 31 human datasets. The evaluation of the analyzed electrode configuration showed that monopolar electrode configurations yield a greater electrode-nerve coupling with a significantly lower stimulus amplitude to activate 80% of the corresponding target nerve at the cost of a lower selectivity compared to the analyzed bipolar electrode configurations. These results verify the described framework as corresponding results were also shown in other studies: Hayden et al. (2011) showed the same effect in both virtual models and *in vivo* experiments for vestibular stimulation in chinchillas. These effects were also previously described in modeling and physiological studies for the stimulation of the auditory nerve in cochlear implants (see for example Bonham and Litvak, 2008 for an overview of related studies and Zhu et al., 2012; Padilla and Landsberger, 2016 for more recent studies comparing the effects of mono-, bi- and tripolar electrode configurations).

Four individual datasets with good quality were chosen from the pool of available labeled vestibular specimen for analyzing the described stimulation scenarios. During the creation of the individual models, careful manual inspection and minimal improvements were performed (if necessary) on the results of each step of the workflow in order to obtain valid meshed geometries for the computation of potential distributions in the inner ear. With the development of an improved preprocessing pipeline for labeled datasets that allows for quality evaluation and (semi-)automatic improvements with special focus on the creation of the electrical model, the analysis of a greater number of individual datasets is planned in future studies. In addition to the individual datasets also the mean shape of a statistical model comprising of 31 vestibular datasets was used for the evaluation of the described vestibular stimulation scenarios by the workflow, defining a representative virtual instance of all individual datasets in the pool.

In the virtual instance describing the mean shape of the statistical model, the nerve branches innervating the otolith organs (N. utricularis and N. saccularis) as well as the cochlea and the IAC were not considered as the main aim of the statistical model described in Fritscher et al. (submitted) is the evaluation of anatomical variations of the bony labyrinth, the nerve branches innervating the cristae of the semicircular canals (N. ampullaris anterior, N. ampullaris lateralis, and N. ampullaris posterior) and the N. facialis. However, our evaluations showed that the statistical model described in Fritscher et al. (submitted) can be successfully coupled with the workflow for evaluation of vestibular stimulation scenarios described here. An extension of the statistical model by considering the nerve branches innervating the otolith organs, the cochlea and the IAC in the statistical model would make it possible to generate additional realistic virtual instances of the human vestibular system that can be used for the analysis of effects of anatomical variations in vestibular stimulation scenarios.

The results summarized in Table 3 show that the orientation of the dipole moment in bipolar electrode configurations does not only influence the selectivity of targeted nerve branch stimulation, but also shows significant differences in the required stimulus amplitude to activate 80% of the corresponding target nerve. These results indicate that deviations in the orientation of an implanted electrode array can have a strong influence on the efficacy, as a higher stimulation current required to activate a target nerve branch also bears the risk of stimulating neighboring non-target nerves. Therefore, special attention should be paid on the electrode orientation when bipolar electrode configurations are considered for vestibular implants. However, *in vivo* studies showed that the brain is able to habituate and adapt to the continuous unnatural stimuli provided by the vestibular implant (high baseline stimulation frequency and simultaneous activation of afferents coincident with the pulses supplied by the vestibular implant) (Lewis, 2016) and that misalignments caused by current spread and imprecise electrode placement can be (partially) compensated by adaptation (van de Berg et al., 2015).

The results obtained from the simulations based on the mean shape of the statistical model are similar compared to the corresponding results of the individual datasets, except for a significantly higher AUC of the monopolar electrode configuration in the N. ampullaris posterior. This difference is caused by the absence of the N. utricularis and N. saccularis in the statistical model, which turned out to be the most sensitive non-target nerves in the monopolar stimulation of the N. ampullaris posterior. For the N. ampullaris anterior and N. ampullaris lateralis, the difference in the AUC for the monopolar stimulation is smaller due to the close proximity of the target nerve branches to other non-target nerve branches present in the statistical model and in the individual models.

A strong similarity was observed when comparing the corresponding fiber recruitment curves of the mean shape obtained by the statistical model, of an individual dataset and the averaged fiber recruitment curves of four datasets. From a qualitative point of view, this result indicates that characteristic effects of the electrode configurations do not depend significantly on anatomical variations. From a quantitative perspective, strong deviations for the stimulus amplitude required to activate 80% of the target nerve were found when comparing the results of the analyzed models. Interestingly, Guinand et al. (2015) also found strong variations in the dynamic range of the applied stimulus amongst others in monopolar, intra-labyrinthine electrode configurations at the ampullary nerves in different patients. The stimulus amplitudes obtained by our simulations to activate 80% of the target nerve were in the same magnitude compared to the dynamic ranges described in their work when considering the same stimulus waveform in our simulations. The longer phase duration of the stimulus (400 μs) in Guinand et al. (2015) caused a slight decrease in the AUC as well as a small decrease in required current to activate 80% of the target nerve in most configurations (data of these additional simulations not shown due to high similarity with data listed in Table 3). Although the patient specific sensitivity to different stimulus amplitudes may be one of the major factors responsible for this outcome, anatomical variations that are also present in the analyzed models may have a significant influence on the required stimulus amplitude to activate the target nerve and on the sensitivity of nearby non-target nerves.

In this work the neurons innervating the otolith organs (N. utricularis and N. saccularis) were considered solely as non-target nerve branches in the simulation scenarios. While the polarity of the hair cells in a crista of a semicircular canal is uniform, the hair cells of the utricle and saccule show varying polarities in most orientations in both the medial and lateral half of the respective macula split by a line of polarity reversal. In the work of Maklad et al. (2010) innervation patterns in the inner ear of mice were analyzed. Their experiments showed that the cristae of the semicircular canals project both to the brainstem and cerebellum. Due to the homogeneous polarity of the hair cells, an overall stimulation of the neurons innervating a targeted crista is feasible. In contrast, Maklad et al. (2010) stated that nearly all vestibular fibers connected to the lateral half of the utricular macula and the vestibular fibers connected to the medial half of the saccular macula project to the cerebellum, while the remaining halves with opposing polarities with respect to their counterparts are innervated by neurons projecting to the brainstem. These complex innervation patterns in the otolith organs make a targeted electrical stimulation emulating horizontal and vertical accelerations a challenging task, which will be considered in our future work.

Compared to the results obtained by Hayden et al. (2011), who used models of the vestibular anatomy of chinchillas in their simulations, significantly higher stimulus amplitudes were required to obtain corresponding fiber recruitment curves, especially in bipolar electrode configurations. This effect can be attributed to anatomical deviations between vestibular systems of humans and chinchillas. The human vestibular anatomy is roughly 2–2.5 times larger compared to the vestibular system of the chinchilla in each linear dimension (Hayden, 2007). The corresponding increase of the diameter of the SCC in the human datasets leads to a lower electrical resistivity in the vestibular lumen due to the significantly lower electrical resistivity of the fluid within the labyrinth compared to the surrounding temporal bone. Consequently, higher stimulus amplitudes are required to obtain corresponding electrical voltages in the human model. Besides the deviations in amplitude, a strong qualitative similarity between the fiber recruitment curves obtained by Hayden et al. (2011) and the results shown in this work was observed. Although the order of magnitude of the stimulus amplitudes required to activate 80% of the target nerve in monopolar electrode configurations is comparable to the dynamic ranges obtained by Guinand et al. (2015), corresponding results of bipolar electrode configurations need to be further verified once appropriate data is available.

In this work selective vestibular target nerve activation was evaluated using intra-labyrinthine mono- and bipolar electrode configurations based on realistic models of the human inner ear. It was found that the transverse parallel bipolar electrode configuration showed the best selectivity among the tested electrode configurations in nearly all analyzed model instances and target nerve branches, while significantly lower activation current thresholds were determined for monopolar configurations at the cost of a lower selectivity. The simulated results also indicate that misaligments of bipolar electrode configurations may require a significantly higher stimulation current to activate the targeted nerve branch. Based on this workflow, a detailed analysis of intra- and extra-labyrinthine electrode configurations with various stimulation waveforms was performed (Schier et al., submitted), making this framework already a valuable tool for current optimization questions of vestibular implants in humans. By continuously extending (e.g., consideration of reactive component of impedance, more realistic anatomical surrounding of the vestibular system) and validating the presented workflow by planned *in vivo* and *in vitro* experiments, additional scientific questions will be addressed and reliably answered in the future, contributing to the ongoing improvement of vestibular prostheses and, consequently, to the health-related quality of life for patients with vestibular disorders.

## Ethics statement

This study was carried out in accordance with the recommendations according to the guidelines of the ethical review committee of the Medical University of Innsbruck with written informed consent from all subjects. All subjects gave written informed consent in accordance with the Declaration of Helsinki. The protocol was approved by the ethical review committee of the Medical University of Innsbruck.

## Author contributions

MH: development of modular workflow, meshing, implementation FEM, experiment design, and evaluation of results. PS: implementation of electrical model and evaluation algorithms, experiment design, and evaluation of results. KF: processing and interpretation of image data, preparation of labeled datasets, and preparation of individual datasets previous to meshing. PR: processing and interpretation of image data, preparation of labeled datasets, and preparation of mean shape of statistical model previous to meshing. LJ: specimen processing, contrast enhancement, manual segmentation of 33 temporal bones and data interpretation. RG: method development for contrast enhancement and imaging, specimen collection over 10 years, specimen preparation, setting of anatomical landmarks, and validation of segmentations. RSa: experiment design, interpretation of data, and organization. RSc: experiment design, and organization. DB: experiment design, conception of the article, interpretation of data, organization. CB: experiment design, conception of the article, and organization. All authors were responsible for drafting and/or revising the manuscript. Additionally, they approved the final version of this document and agreed upon the accountability for all aspects of this work.

### Conflict of interest statement

RSa works as a research engineer for MED-EL GmbH in Innsbruck, Austria. The other authors declare that the research was conducted in the absence of any commercial or financial relationships that could be construed as a potential conflict of interest.
